# Supplementary material for: Rapid diagnostic tests and ELISA for diagnosing chronic Chagas disease: Systematic revision and meta-analysis
Source: PLoS Negl Trop Dis. 2022 Oct 18;16(10):e0010860. doi: 10.1371/journal.pntd.0010860 (PMC9616215; doi:10.1371/journal.pntd.0010860)
Supplement: S1 Database — (DOCX) [file pntd.0010860.s003.docx]

**S1 Database**

**Database search strategy**

**PUBMED**

("Chagas Disease"[MeSH] OR "Trypanosoma cruzi"[MeSH] AND (ELISA OR (enzyme AND linked AND assay) OR (rapid diagnosis test OR rapid test))) AND (sensitiv*[Title] OR sensitivity and specificity[MeSH Terms] OR diagnos*[Title/Abstract] OR diagnosis[MeSH:noexp] OR diagnosis, differential[MeSH:noexp] OR diagnosis[Subheading:noexp] OR "Reproducibility of Results"[Mesh] OR reliability OR reproducibility OR accuracy) AND ("last 10 years"[PDat])

**LILACS**

tw:(((tw:("Chagas Disease")) OR (mh:(trypanosoma cruzi))) AND ( (tw: (rapid diagnosis test)) OR ((tw: (rapid test)) OR ((tw: (rapid diagnosis))))) OR (tw:(elisa) OR ((tw:(enzimi AND linked AND and assay)) OR (serodiagnostic test))) AND ((tw: (sensitiv*)) OR ((mh sensitivity)) OR ((mh specificity))) OR ((tw: (diagnos*)) OR (mh:(diagnosis)) OR (mh:( diagnosis differential)) ) OR ((mh:(reproducibility of results)) OR (tw:(reliability)) OR ((mh:(data accuracy)) OR (tw:(accuracy))))) AND ( db:("LILACS") AND mj:("Enfermedad de Chagas" OR "Trypanosoma cruzi" OR "Pruebas Serológicas" OR "Ensayo de Inmunoadsorción Enzimática" OR "Pruebas Inmunológicas" OR "Sensibilidad y Especificidad") AND la:("es" OR "en" OR "pt"))

**SCOPUS**

( TITLE-ABS-KEY ( chagas  AND  disease )  OR  TITLE-ABS-KEY ( trypanosoma  AND  cruzi )  OR  TITLE-ABS-KEY ( chronic  AND  chagas  AND  disease )  OR  TITLE-ABS-KEY ( trypanosoma  AND  cruzi  AND  infection )  AND  TITLE-ABS-KEY ( enzyme-linked  OR  immunosorbent  OR  assay )  AND  TITLE-ABS-KEY ( rapid  OR  diagnosis  OR  test )  AND  TITLE-ABS-KEY ( sensitivity )  OR  TITLE-ABS-KEY ( diagnos* )  OR  TITLE-ABS-KEY ( reproducibility  AND  of  AND  results )  AND  TITLE-ABS-KEY ( humans )  AND NOT  TITLE-ABS-KEY ( animals ) )  AND  ( LIMIT-TO ( PUBYEAR ,  2020 )  OR  LIMIT-TO ( PUBYEAR ,  2019 )  OR  LIMIT-TO ( PUBYEAR ,  2018 )  OR  LIMIT-TO ( PUBYEAR ,  2017 )  OR  LIMIT-TO ( PUBYEAR ,  2016 )  OR  LIMIT-TO ( PUBYEAR ,  2015 )  OR  LIMIT-TO ( PUBYEAR ,  2014 )  OR  LIMIT-TO ( PUBYEAR ,  2013 )  OR  LIMIT-TO ( PUBYEAR ,  2012 )  OR  LIMIT-TO ( PUBYEAR ,  2011 )  OR  LIMIT-TO ( PUBYEAR ,  2010 ) )  AND  ( LIMIT-TO ( LANGUAGE ,  "English" )  OR  LIMIT-TO ( LANGUAGE ,  "Spanish" )  OR  LIMIT-TO ( LANGUAGE ,  "Portuguese" ) )

**Web of Science:**

(TS=(Chagas AND Disease)

OR TS=(Trypanosoma AND cruzi)

OR TS=(Trypanosoma AND cruzi) )

AND  (TS=(ELISA) OR TS=(enzyme AND linked AND assay)

OR TS=(enzyme-linked AND immunosorbent AND assay))

AND ( TS=(rapid AND diagnos* AND test) OR TS=(serological AND test))

AND (TS=diagnos OR TS=sensitiv

OR TS=sensitivity OR TS=specificity

OR TS=(Reproducibility AND Results)

OR TS=diagnosis OR TS=differential

OR TS=reliability OR TS=reproducibility OR TS=accuracy)
