# Supplementary material for: Rapid diagnostic tests and ELISA for diagnosing chronic Chagas disease: Systematic revision and meta-analysis
Source: PLoS Negl Trop Dis. 2022 Oct 18;16(10):e0010860. doi: 10.1371/journal.pntd.0010860 (PMC9616215; doi:10.1371/journal.pntd.0010860)
Supplement: S1 Table — (DOCX) [file pntd.0010860.s004.docx]

**S1 Table.** ELISA test. Bivariate model goodness of fit for choosing the basic features: link, random effect distribution for the bivariate model

| Model | Link.function | model | DIC |
| --- | --- | --- | --- |
| Binomial+Normal | logit | Bivariate | 630 |
| Binomial+Normal | probit | Bivariate | 631 |
| Binomial+Scale mixture | probit | Bivariate | 633 |
| Binomial+Scale mixture | cloglog | Bivariate | 636 |
| Binomial+Scale mixture | logit | Bivariate | 638 |
| Binomial+Normal | cloglog | Bivariate | 638 |
