# Supplementary figures and images for: Rapid diagnostic tests and ELISA for diagnosing chronic Chagas disease: Systematic revision and meta-analysis
Source: PLoS Negl Trop Dis. 2022 Oct 18;16(10):e0010860. doi: 10.1371/journal.pntd.0010860 (PMC9616215; doi:10.1371/journal.pntd.0010860)

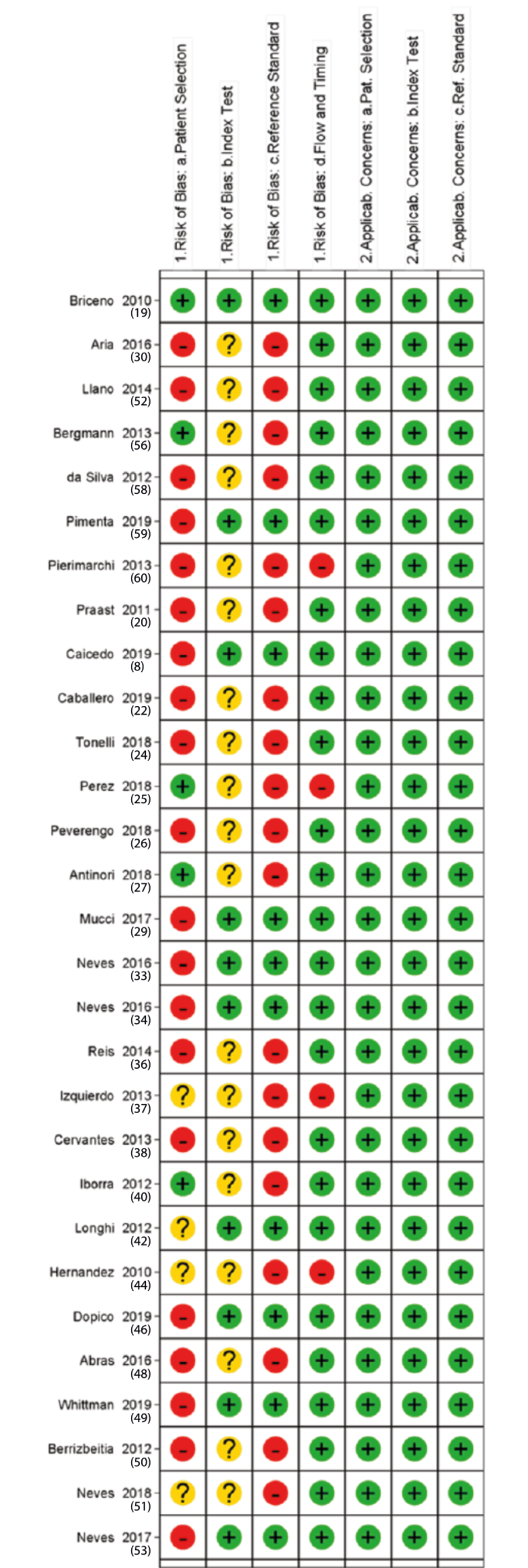

Supplement: S1 Fig — (TIFF) [file pntd.0010860.s006.tiff]

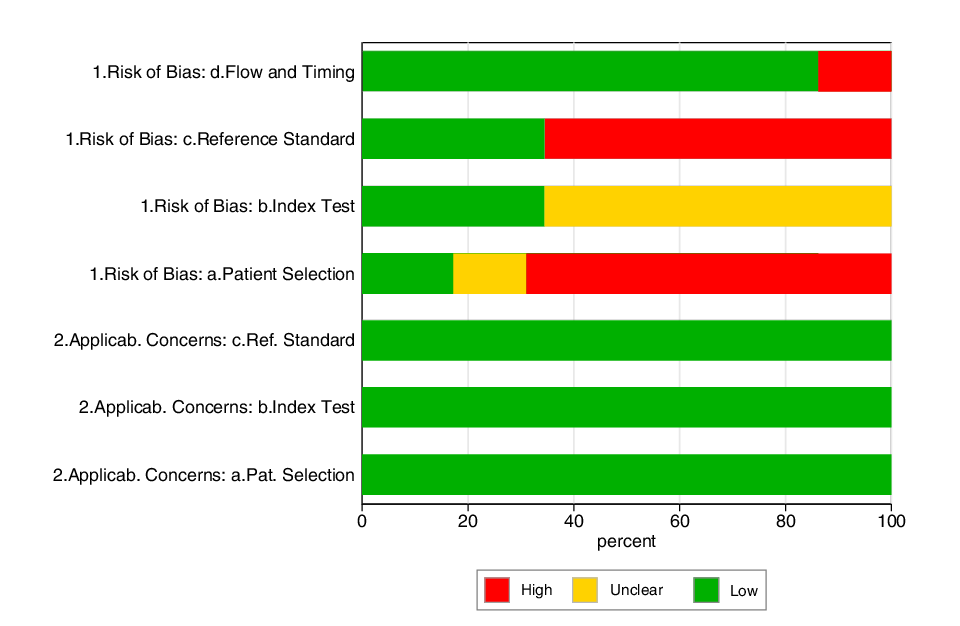

Supplement: S2 Fig — (TIFF) [file pntd.0010860.s007.tiff]

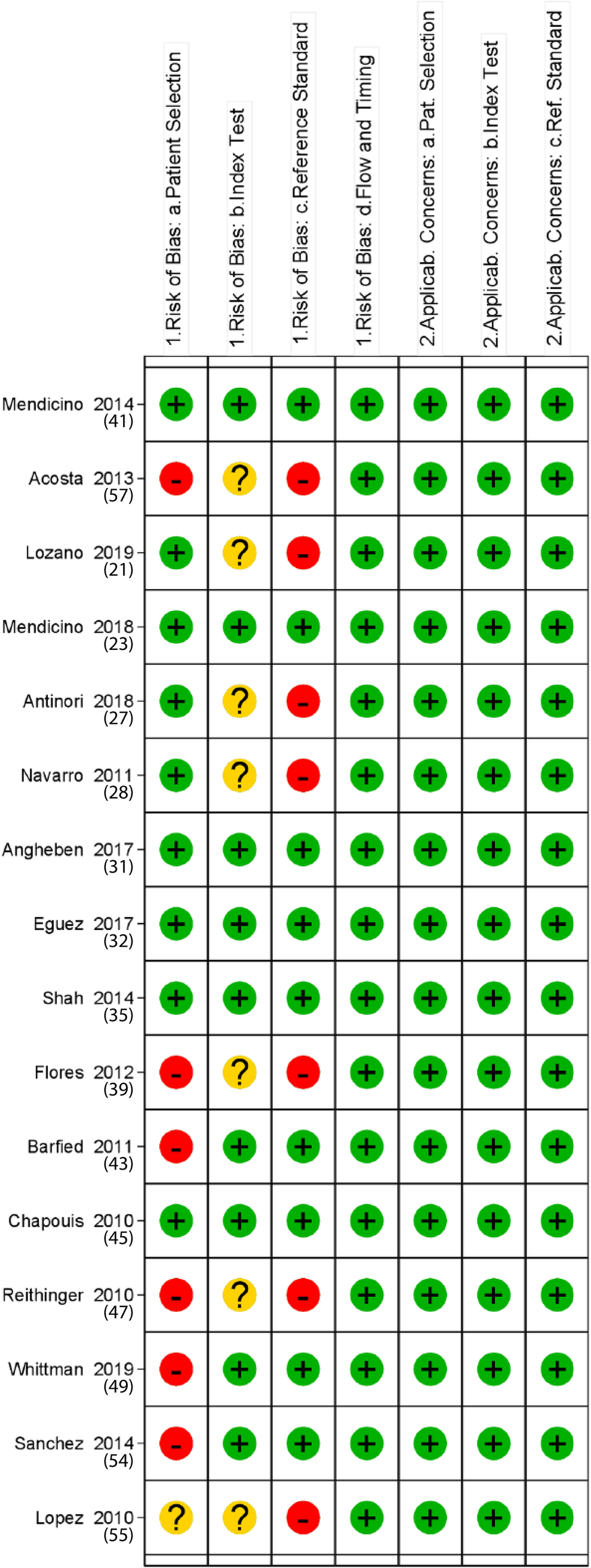

Supplement: S3 Fig — (TIFF) [file pntd.0010860.s008.tiff]

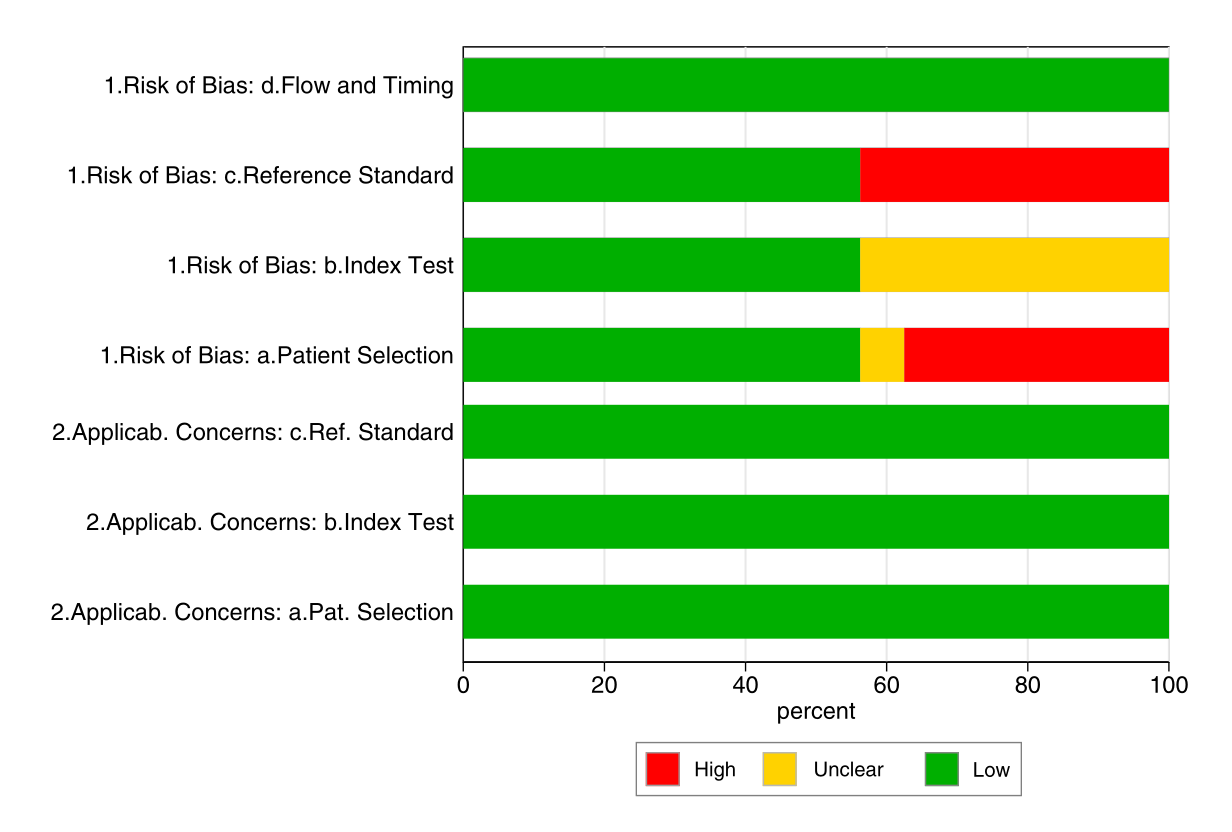

Supplement: S4 Fig — (TIFF) [file pntd.0010860.s009.tiff]

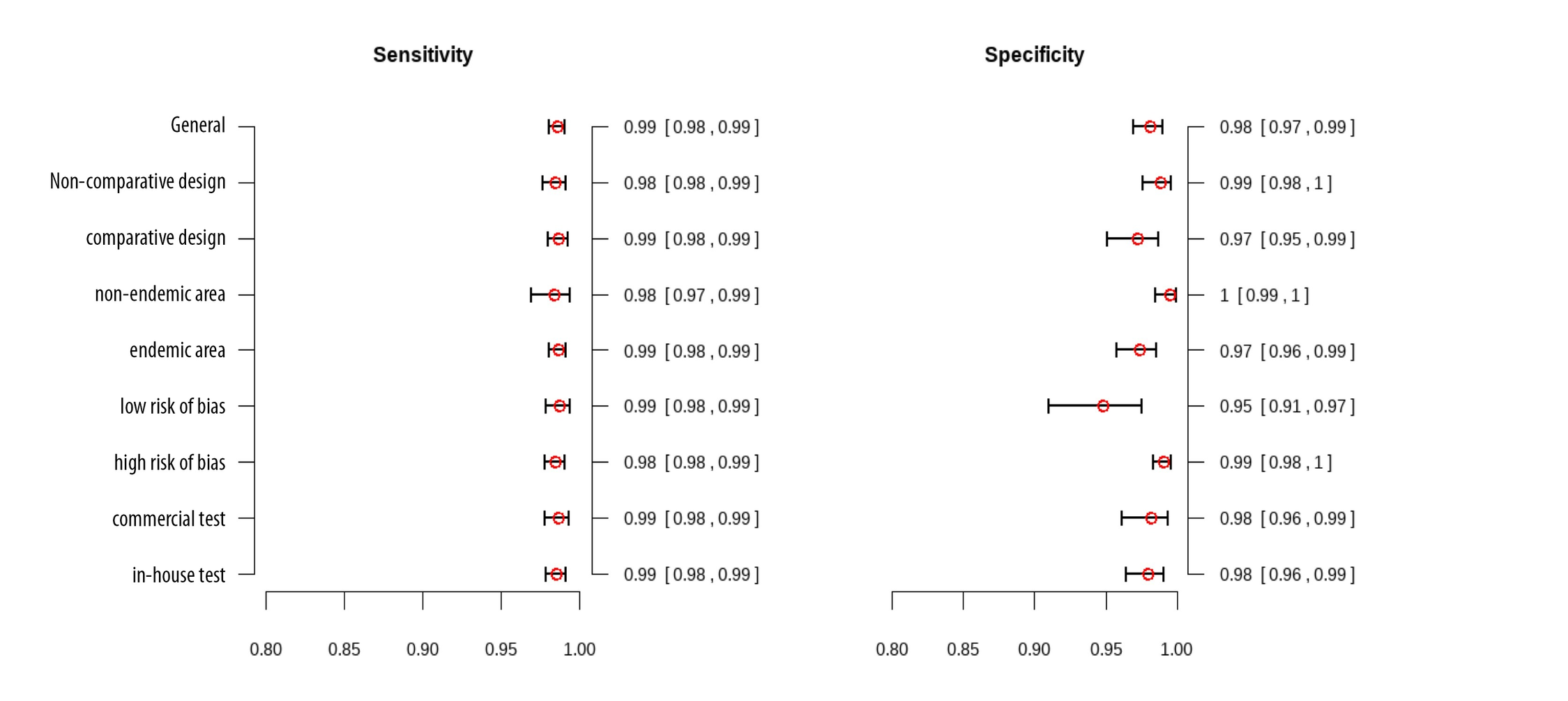

Supplement: S5 Fig — (TIFF) [file pntd.0010860.s010.tiff]

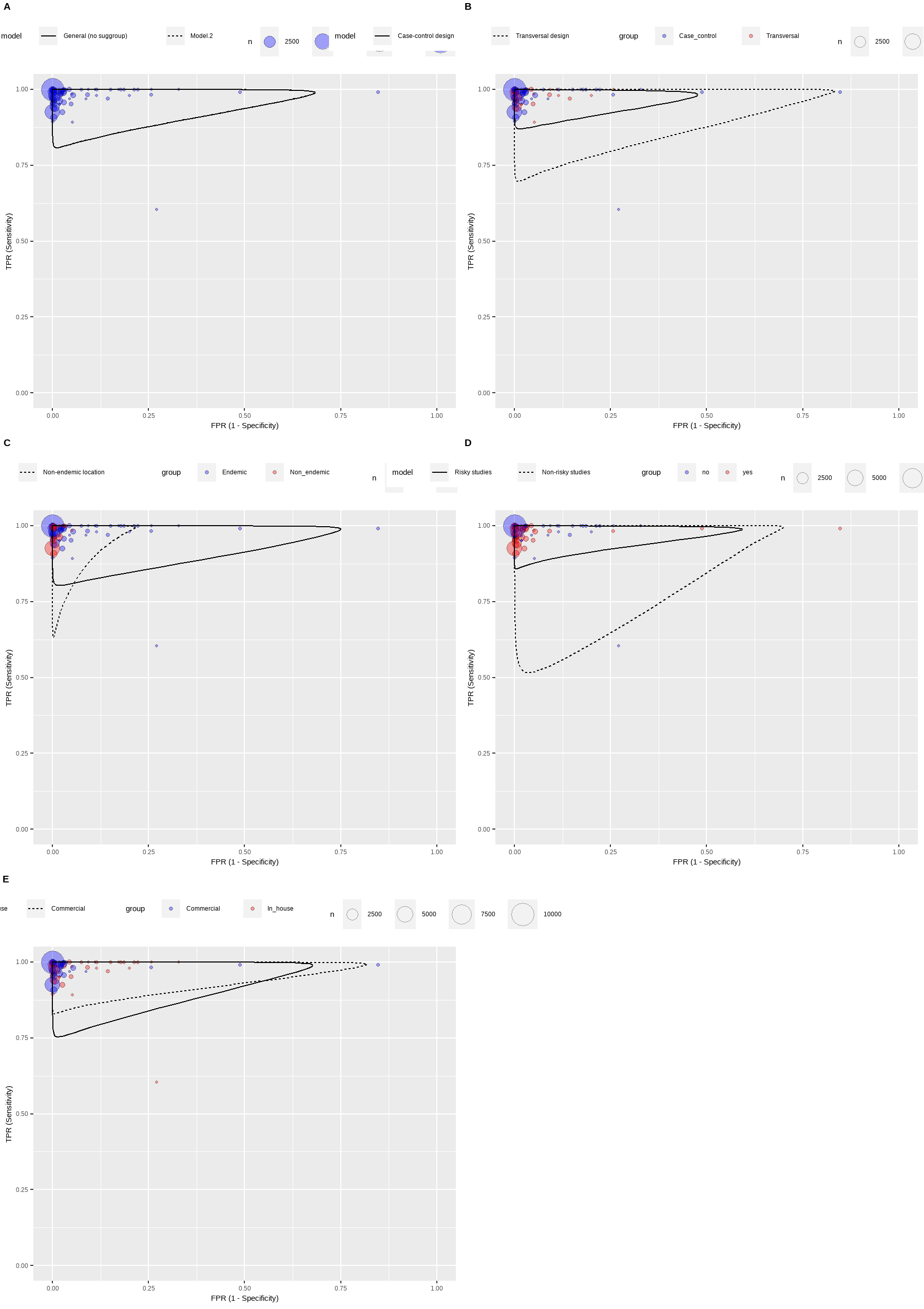

Supplement: S6 Fig — (TIFF) [file pntd.0010860.s011.tiff]

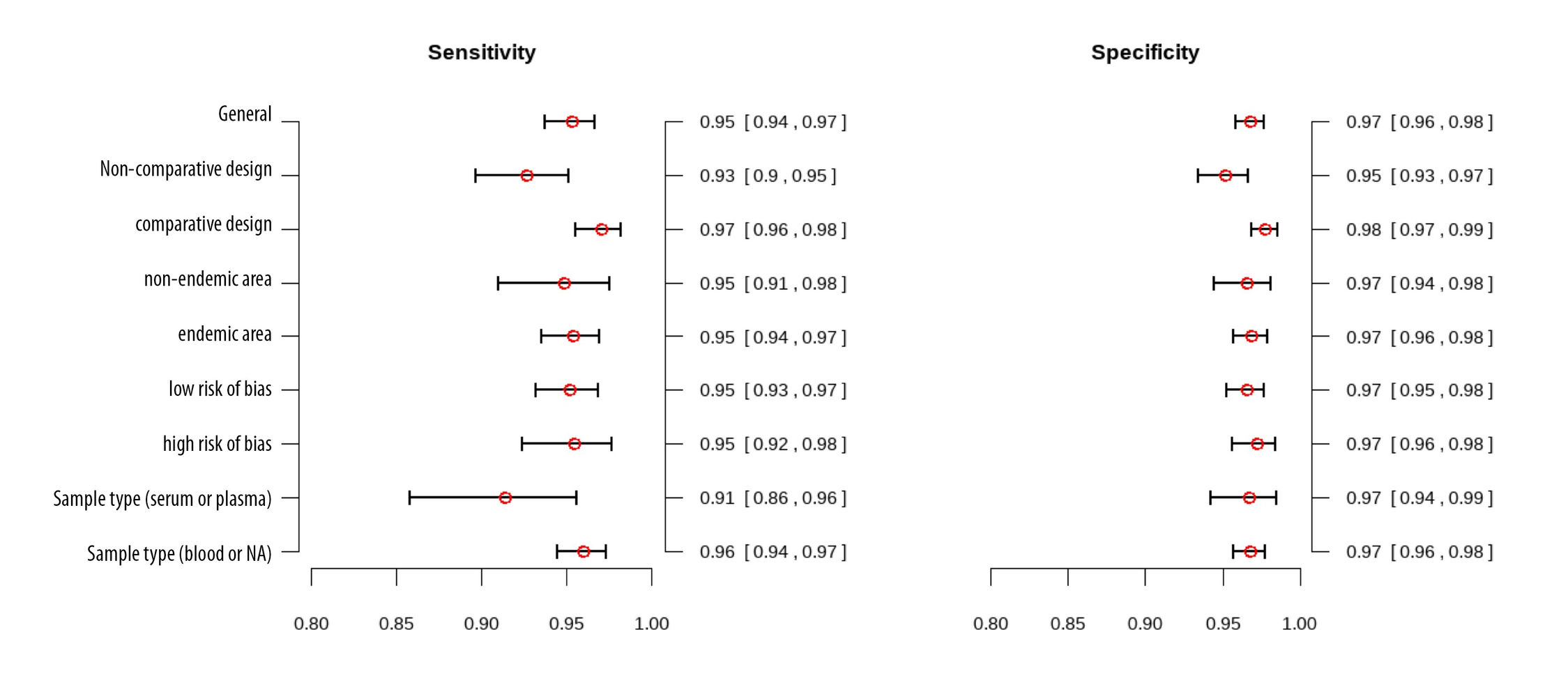

Supplement: S7 Fig — (TIFF) [file pntd.0010860.s012.tiff]

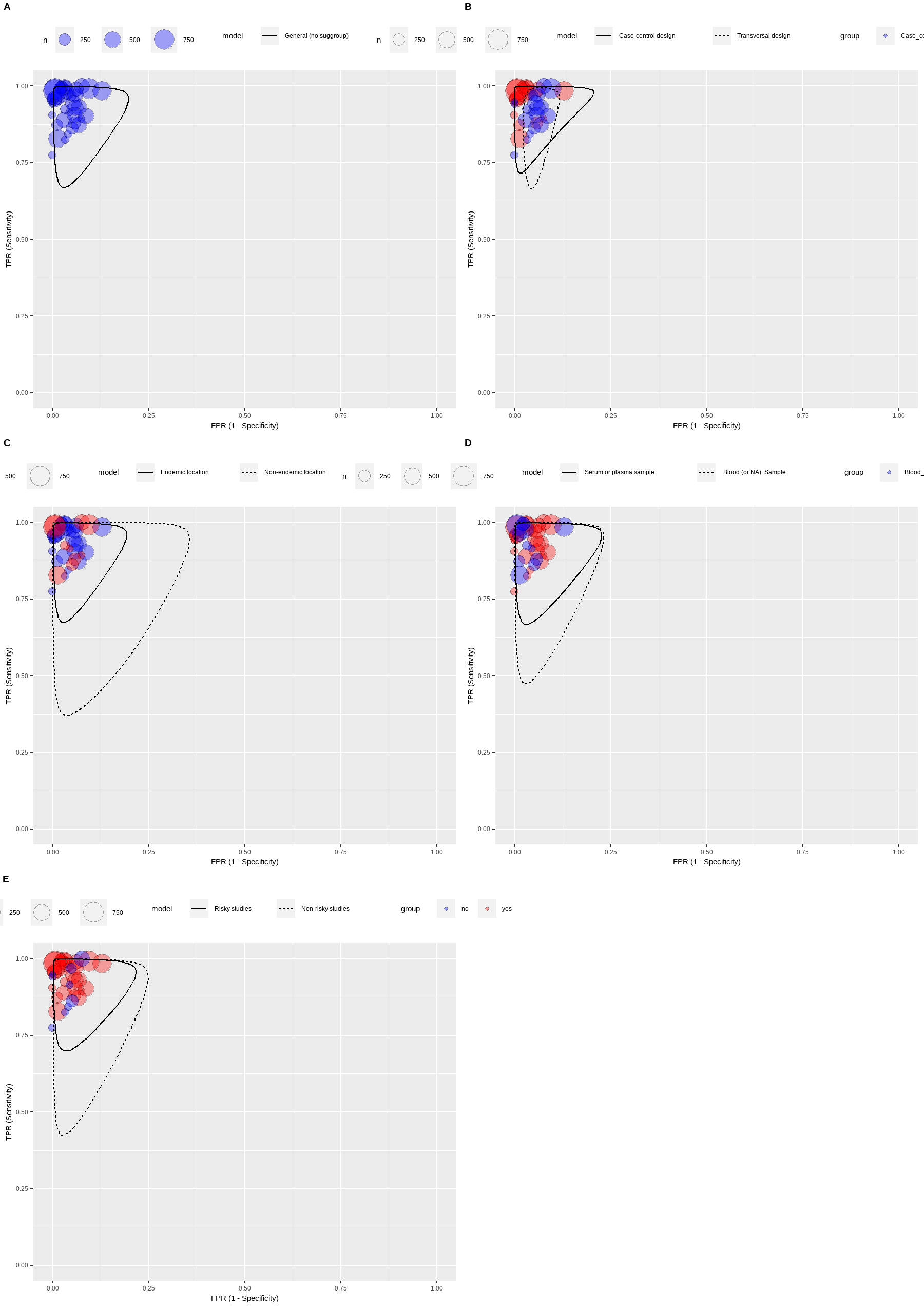

Supplement: S8 Fig — (TIFF) [file pntd.0010860.s013.tiff]

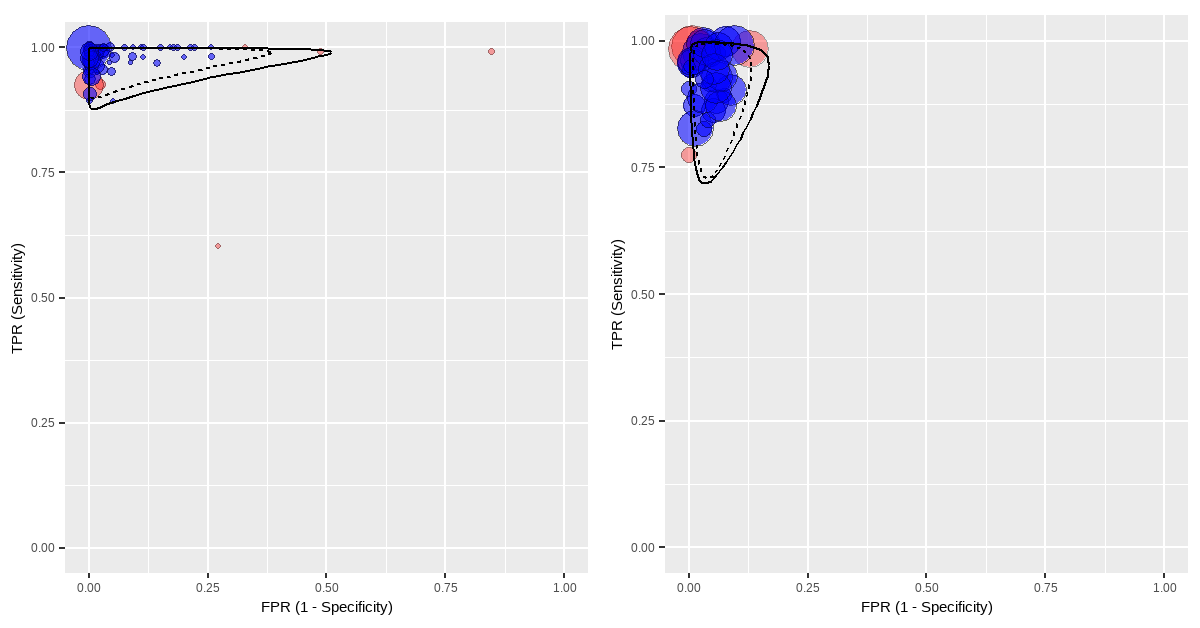

Supplement: S9 Fig — Predictive region in the global ROC space and after excluding the most influential studies for ELISA (A) and RDTs (B). (TIFF) [file pntd.0010860.s014.tiff]
