# Supplementary material for: Rapid diagnostic tests and ELISA for diagnosing chronic Chagas disease: Systematic revision and meta-analysis
Source: PLoS Negl Trop Dis. 2022 Oct 18;16(10):e0010860. doi: 10.1371/journal.pntd.0010860 (PMC9616215; doi:10.1371/journal.pntd.0010860)
Supplement: Attachment — Submitted filename: Letter.docx [file pntd.0010860.s015.docx]

August 27^th^, 2022

To: Editors

**PLOS Neglected Tropical Diseases**

RE: Article Review PNTD-D-22-00624: “Rapid Diagnostic Tests and ELISA for diagnosing Chronic Chagas Disease: Systematic revision and meta-analysis”. “Chagas Disease Diagnosis: Systematic revision and meta-analysis”.

First, the authors would like to thank the reviewers and the editors for their clear and concise comments. It is important to note that our corrections were made following the reviewer’s recommendations.

We hope we have addressed the highlighted problems. We are thankful for their time and attention. Below you will find our responses to their evaluations.

Sincerely,

**Sandra Suescún-Carrero**

Corresponding author

The authors present a detailed systematic review and meta-analysis of studies assessing the test performance of ELISAs and RDTs for the diagnosis of chronic Chagas Disease. The methodology is well designed and the results have some clinical applicability. However, presentation of the results could be improved to enhance the flow and structure of the authors' findings.

Answers to questions posed by reviewers.

----------------------------------------------------------------------------------------

**Methods**

**Reviewer #1**: The objectives of the study are clearly articulated with a clear testable hypothesis stated. The study design is appropriate to address the stated objectives.

1) The Abstract mentions that the search was performed for studies published between May and August 2020, but the search was performed between May and August 2020, and included studies published between 2010 and 2020.

*Answer*: We accept the reviewer’s recommendation.

The following changes were made: “Published between May and August 2020” is changed to “published between 2010 and 2020” on (L 30).

2) In Eligibility Criteria, the authors said that “The search included studies that estimated sensitivity, specificity, and predictive values…”. However, the predictive values have not been shown or analyzed in the Results.

*Answer*: We accept the reviewer’s recommendation.

The following changes were made: “The search included studies that estimated sensitivity, specificity, and predictive values of ELISA or RDT index tests for chronic CD” it is changed to “The search included studies that estimated sensitivity and specificity of ELISA or RDT index tests for chronic CD”, omitting the words: predictive values (L 116 to 117).

3) Definitions for data extraction. The presentation of the different groups to classify subjects is confusing and individuals may be in different groups. I believe that this classification could change to endemic/non-endemic region without losing relevant information.

*Answer*: We accept the reviewer’s recommendation.

The paragraph below:

The subjects included in the different studies were classified as: patients with suspected chronic CD (if they had lived or lived in an endemic area for CD and were exposed to any of its risk factors), participants in screening programs for the disease (people who attend primary health care programs for the diagnosis of chronic CD), blood donors (individuals who go to blood banks and undergo mandatory screening tests for different infectious agents), and the general population (individuals who agreed to participate in studies of research centers, health care institutions or public health laboratories).

Was replaced by the paragraph:

The subjects included in the different studies were classified into: patients who had lived or resided in an endemic area for CD and patients who reside in a non-endemic area. (L 149 to 150).

4) Especially for RDT, it might be relevant to analyze whether the test was performed by skilled or unskilled health workers.

Information related to the reading of the processing of the RDTs is included.

Of the 16 studies that analyzed RDTs, 8 did not report who read the results (23,26,36,37,40,54,60,61), 4 did not specify whether or not they had training in reading this type of test (10,47,50,56) and 4 studies indicated that the reading was done by qualified personnel (32,39,43,52) (L 273 to 275).

**Reviewer #2:** (No Response)

**Reviewer #3:**

-Is the study design appropriate to address the stated objectives? NEUTRAL. Please refer to commentary below.

1) The objective of the study is clearly indicated in the manuscript, although in the abstract please include the time lapse of publication (10 years) that was considered to include the studies (as this information if more important for the potential reader) instead of the time (May to August 2020) that the search was carried out.

This was the same observation made by Reviewer #1.

*Answer*: We accept the reviewer’s recommendation.

The following changes were made: “Published between May and August 2020” is changed to “published between 2010 and 2020” on (L 30).

------------------------------------------------------------------------------------------------

**Results**

**Reviewer #1**: The analysis presented match the analysis plan, the results are clearly and completely presented. The figures are of sufficient quality for clarity.

*Answer*: Thank you for the comment.

**Reviewer #2**: (No Response)

**Reviewer #3:** -Does the analysis presented match the analysis plan? YES

1) Are the results clearly and completely presented? YES. Although it is advisable to review English as some phrases have a construction that can be confusing for the reader.

*Answer*: We accept the reviewer’s recommendation.

The paragraph below:

In total, 33 articles carried out various substudies (76 for ELISA tests and 39 for RDTs), with more than one test using different peptides, studies carried out in several countries, different reference laboratories or evaluation of several index tests, which data are shown and analyzed separately in this research.

Was replaced by the paragraph:

In 33 articles (76 for ELISA tests and 39 for PDR), several substudies included aspects such as tests with different peptides, populations from different countries, participation of several reference laboratories or evaluation of different index tests. This data is presented and analyzed by separated in the present investigation (L 249 to 252).

In the results, the redaction was revised (L 229 to 494).

------------------------------------------------------------------------------------------------

**Conclusions**

**Reviewer #1**: The conclusions are supported by the data presented; the limitations of analysis are clearly described; the authors discuss how these data can be helpful to advance our understanding of the topic under study.

*Answer*: Thank you for the comment.

**Reviewer #2:** (No Response)

**Reviewer #3:**

-Are the conclusions supported by the data presented? YES
-Are the limitations of analysis clearly described? YES
-Do the authors discuss how these data can be helpful to advance our understanding of the topic under study? YES
-Is public health relevance addressed? YES

*Answer*: Thank you for the comment.

-------------------------------------------------------------------------------------------------

**Editorial and Data Presentation Modifications**?

**Reviewer #1**:

1)Reference 20 does not follow the required format for Plos NTD.

*Answer*: We accept the reviewer’s recommendation.

The reference number 20 was corrected

The reference below:

20. JJ D, P M, L I. The performance of tests of publication bias and other sample size effects in systematic reviews of diagnostic test accuracy was assessed. J Clin Epidemiol. 2005;58:882-93. https://doi.org/10.1016/J.JCLINEPI.2005.01.016

Was replaced by the reference:

20. J Deeks JJ, Macaskill P, Irwig L. The performance of tests of publication bias and other sample size effects in systematic reviews of diagnostic test accuracy was assessed. J Clin Epidemiol. 2005;58(9):882-93. <https://doi.org/10.1016/J.JCLINEPI.2005.01.016> (L 608 to 610).

**Reviewer #2**: (No Response)

**Reviewer #3**: (No Response)

--------------------------------------------------------------------------------------------------

**Summary and General Comments**

**Reviewer #1**: Regarding the study entitled “Rapid Diagnostic Tests and ELISA for diagnosing Chronic Chagas Disease: Systematic revision and meta-analysis”, I think the authors have done a good work. The diagnosis of Chagas disease, particularly in the chronic phase, is based on the detection of specific IgG antibodies against Trypanosoma cruzi, with the ELISA test being the most common in laboratories, and immunochromatographic tests or rapid diagnostic tests (RDT) the most common in primary health care centers or field screening studies. In this framework, the authors summarize the available evidence on the diagnostic validity of ELISA and RDT in individuals with suspected chronic Chagas disease. The manuscript is easy to read and the results are straightforward. The conclusions of the work are relevant for public health. However, minor corrections are needed to make the article fit for publication in the Plos NTD.

*Answer*: Thank you for the comment.

**Reviewer #2:** Authors perform a systematic review and meta-analysis of studies assessing the sensitivity and specificity of enzyme-linked immunosorbent assay (ELISA) and Rapid Diagnostic Tests (RDT) among individuals with suspected chronic Chagas Disease. The methodology is strong and well designed with interesting results that would have some clinical applicability. The display of the results can be organized better to enhance the flow and structure of the information:

1)Paper has too many figures. Please choose 3-4 to include in the main paper and move the rest to the supplemental data, especially the quality assessment data

*Answer*: We accept the reviewer’s recommendation.

Decreasing the number of figures from 11 to 5.

The following paragraph:

The quality assessment of the studies included in the analysis of ELISA tests is shown in Figures 2 and 3; and those of RDTs are shown in Figures 4 and 5

Was replaced:by the paragraph:

The quality assessment of the studies included in the analysis of ELISA tests is shown in Figures S1 and S2; and those of RDTs are shown in Figures S3 and S4. (L 302 to 303)

The legends of figures 2, 3, 4 and 5 were deleted.

Figure 2. Risk of bias and applicability of ELISA tests.

Figure 3. Summary of risk of bias and applicability of ELISA tests.

Figure 4. Risk of bias and applicability of RDTs

Figure 5. Summary of risk of bias and applicability of RDTs

Figure 6 becomes Figure 2. Sensitivity and specificity of ELISA tests in the studies included in the meta-analysis (L 334)

Figure 7 becomes Figure 3. Predictive region in the ROC space, with all the studies for ELISA tests (L 337)

Figure 6 becomes Figure 4. Sensitivity and specificity of Rapid Diagnostic Tests in the studies included in the meta-analysis (L 346)

Figure 7 becomes Figure 5. Predictive region in the ROC space, with all the studies for RDTs (L 350)

6 figures (2, 3, 4, 5, 10 and 11) are transferred to “Supporting information”, being numbered as follows: (S4 Fig, S5 Fig, S6 Fig, S7 Fig, S8 Fig, S9 Fig).

2)Key tables were moved to the supplement. Paper needs a table 1 with all the important information from all studies. Tables s4, s5, and s6 need to appear in the main manuscript as a single table or 2 tables. Please incorporate as much info as possible

*Answer*: We accept the reviewer’s recommendation.

Tables S4, S5 and S6 that were in Supporting information are transferred to the main manuscript, leaving them in a single table, called table 1 (L 256). Taking into account the above, the Supporting information was adjusted as follows:

The following paragraph:

Supporting information

**S1 Checklist.** PRISMA-DTA for abstracts checklist. (DOCX)

**S2 Checklist.** PRISMA-DTA checklist. Checklist for reporting of systematic reviews and meta-analysis of diagnostic test accuracy studies. (DOC). (DOCX)

**S3 Database search strategy.** (DOCX)

**S4 Table.** Qualitative analysis of studies selected - ELISA assays (DOCX)

**S5 Table.** Qualitative analysis of studies selected – RDT assays (DOCX)

**S6 Table.** Qualitative analysis of studies selected - ELISA and RDT assays (DOCX)

**S7 Table.** ELISA test. Bivariate model goodness of fit for choosing the basic features: link, random effect distribution for the bivariate model (DOCX)

**S8 Table.** PDR. Bivariate model goodness of fit for choosing the basic features: link, random effect distribution for the bivariate model (DOCX)

**S9 Fig.** Predictive region in the global ROC space and after excluding the most influential studies for ELISA (A) and RDTs (B) (TIFF)

Was replaced by the paragraph:

Supporting information

**S1 Checklist.** PRISMA-DTA for abstracts checklist. (DOCX)

**S2 Checklist.** PRISMA-DTA checklist. Checklist for reporting of systematic reviews and meta-analysis of diagnostic test accuracy studies. (DOC). (DOCX)

**S3 Database search strategy.** (DOCX)

**S4 Table.** ELISA test. Bivariate model goodness (DOCX)

**S5 Table.** RDTs Bivariate model goodness (DOCX)

**S1 Fig.** Risk of bias and applicability of ELISA tests (TIFF)

**S2 Fig.** Summary of risk of bias and applicability of ELISA tests (TIFF)

**S3 Fig.** Risk of bias and applicability of RDTs (TIFF)

**S4 Fig.** Summary of risk of bias and applicability of RDTs (TIFF)

**S5 Fig.** Estimated sensitivity and specificity of ELISA tests by subgroups (TIFF)

**S6 Fig.** Predictive region of ELISA tests by subgroups (TIFF)

**S7 Fig.** Estimated sensitivity and specificity of RDTs by subgroups (TIFF)

**S8 Fig.** Predictive region of RDTs by subgroups (TIFF)

**S9 Fig.** Predictive region in the global ROC space and after excluding the most influential studies for ELISA (A) and RDTs (B) (TIFF)

3)Even though it was stated in the method section, no information was provided for the heterogeneity and I^2^ scores data in the text. Please include and discuss

Information related to the heterogeneity of ELISA tests was included, therefore, it is observed that there is greater heterogeneity in specificity than in sensitivity (L 332).

Information related to the heterogeneity of the RDTs was included, therefore, it is identified that there is a slightly greater heterogeneity for sensitivity (L 341 to 342).

Information related to the heterogeneity of ELISA tests and RDTs was included in the discussion.

Regarding heterogeneity, in the ELISA tests it was identified that this measure was higher in specificity than in sensitivity, which is similar to that reported by Brazil (64), while in the RTDs, the heterogeneity was limited, being slightly higher for sensitivity, coinciding with what was described in the systematic review carried out by Angheben (39), results that differ from those reported by Afonso (65) (L 405 to 408).

We did not include the use of I^2^ to determine heterogeneity within the statistical analysis, since this statistic is more used to assess the heterogeneity of a single outcome variable, it may not be informative or significant by itself when analyzing studies of diagnostic tests that analyze two variables (sensitivity and specificity), therefore it must be interpreted with caution because it does not consider a threshold effect, which is one of the most important sources of heterogeneity in studies of diagnostic tests. Therefore, for the evaluation of heterogeneity in the present meta-analysis, we used the inspection of forest plots of sensitivity and specificity, together with visual estimation of the ROC diagram, in addition to the use of a bivariate random-effects model.

4)Subgroup analysis did not provide p-values for each analysis. Where the different subgroups statistically different? Was the heterogeneity better with each of those subgroups?

*Answer*: We accept the reviewer’s recommendation.

The p-value was not included in the analyzes presented as it was not part of the purpose of the study.

Information related to the heterogeneity of the subgroups of the ELISA tests was included.

Regarding the ELISA tests, the moderators that showed the greatest difference in the predictive region were study design (clinical comparative or non-comparative), where the comparative studies presented low heterogeneity, while the non-comparative studies had greater heterogeneity; and the study risk subgroup (low or high risk of bias), because the studies are similar when they present risk, showing a lower precision (Figure S6) (L 359 to 363)

Information related to the heterogeneity of the RDTs subgroups is included.

Regarding the RDTs, the moderators that show the greatest difference in the predictive region are the subgroups study area (endemic or non-endemic), sample type (serum, whole blood or not applicable), and study risk (low or high risk of bias) (Figure S8) (L 373 to 375).

We include the ROC space figures of the ELISA test subgroups and RDTs in supplementary material (Figure S6 and Figure S8) (L 363 and 375).

5) Line 342: sentence: "There were studies with atypical values (10,40,54) (Figure 8)" what atypical means? Please clarify

*Answer*: We accept the reviewer’s recommendation.

The following paragraph:

There were studies with atypical values

Was replaced: by the paragraph:

There were studies with atypical values or outliers (Those that did not follow the patron of most of the studies, which means that they were strayed of the tendency) (L 341 to 342).

**Reviewer #3**: (No Response)

--------------------------------------------------------------------------------------------------------------

**Figure Files:**

Answer: All figures were uploaded to the PACE tool.

**Data Requirements**

Answer: The data used for development of the article is included in the support materials.

**Reproducibility**

Answer: The protocol was deposited in protocol.io

Identifier (DOI): <http://dx.doi.org/10.17504/protocols.io.4r3l2oxppv1y>

**References**

Answer: Review of the list of references
